# Small RNAs in Plant Responses to Abiotic Stresses: Regulatory Roles and Study Methods

**DOI:** 10.3390/ijms161024532

**Published:** 2015-10-15

**Authors:** Yee-Shan Ku, Johanna Wing-Hang Wong, Zeta Mui, Xuan Liu, Jerome Ho-Lam Hui, Ting-Fung Chan, Hon-Ming Lam

**Affiliations:** 1 Center for Soybean Research of State Key Laboratory of Agrobiotechnology and School of Life Sciences, The Chinese University of Hong Kong, Shatin, Hong Kong; E-Mails: ysku@ymail.com (Y.-S.K.); hang0418@gmail.com (J.W.-H.W.); zeta.mui@gmail.com (Z.M.); jeromehui@cuhk.edu.hk (J.H.-L.H.); tf.chan@cuhk.edu.hk (T.-F.C.); 2 Department of Computer Science, The University of Hong Kong, Pokfulam, Hong Kong; E-Mail: liuxuan@connect.hku.hk

**Keywords:** abiotic stress, bioinformatics, microRNA, small RNA, transcriptional regulation

## Abstract

To survive under abiotic stresses in the environment, plants trigger a reprogramming of gene expression, by transcriptional regulation or translational regulation, to turn on protective mechanisms. The current focus of research on how plants cope with abiotic stresses has transitioned from transcriptomic analyses to small RNA investigations. In this review, we have summarized and evaluated the current methodologies used in the identification and validation of small RNAs and their targets, in the context of plant responses to abiotic stresses.

## 1. Introduction: The Importance of Small RNAs

**Figure 1:**
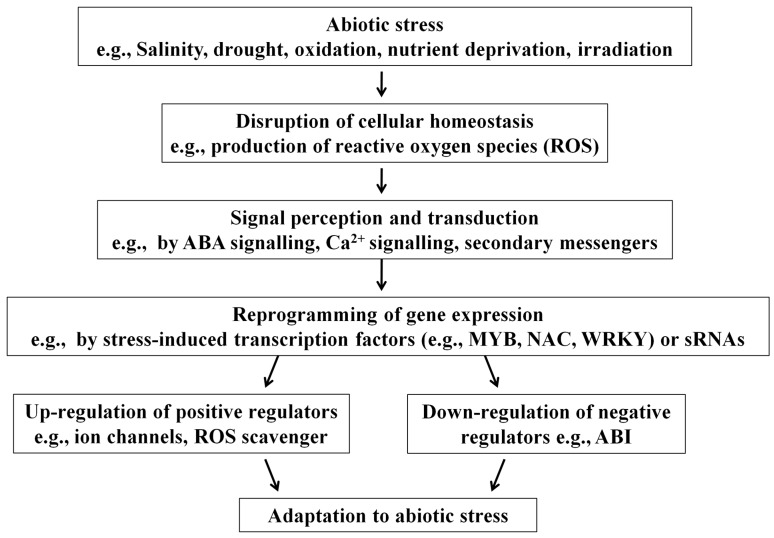
A simplified representation to illustrate the central role of gene expression reprogramming in triggering the adaption to abiotic stresses. Upon abiotic stresses, cellular homeostasis is disrupted. The signal is sensed and transduced by signaling molecules. This brings forth the reprogramming of gene expression which involves transcriptional factors and sRNAs, resulting in the up-regulation of positive regulators and down-regulation of negative regulators.

Plants are constantly challenged by environmental abiotic stresses such as high salinity, drought, flooding, extreme temperatures, and high irradiation. These adverse effects hamper plant growth and development, and may even lead to premature death. To combat the changes in the environment, plants trigger a network of genetic regulations to turn on protective mechanisms. This involves reprogramming of gene expressions. The expressions of protective genes are up-regulated while those of negative regulators are down-regulated. Transcriptional reprogramming (Figure 1) is one essential step to trigger the adaptation processes [1,2,3]. On the other hand, increasing evidence suggests that small RNAs (sRNAs) play important roles in the regulation of gene expressions. High-throughput sequencing and computational prediction have been important tools for identifying abiotic stress-related sRNAs. Guided by computational prediction, experimental validation and functional tests are required to understand the roles of the identified sRNAs. In this review, we will summarize the current findings of the regulatory roles of sRNAs of plants under abiotic stresses as well as the computational and experimental methods used in sRNAs studies.

sRNAs are short transcripts (21–25 nucleotides) that do not translate into proteins, but instead regulate vital biological processes and epigenetic events. The first group of sRNAs discovered are characterized by their short and unique lengths and their participation in post-transcriptional gene silencing (PTGS) in plants such as tobacco, *Arabidopsis* and tomato [4,5,6]. These regulators were later referred to as small interfering RNAs (siRNAs). Efforts to clone dicer cleavage products in the model plant *Arabidopsis* initiated the massive discovery of sRNAs in plants, with many of them sharing characteristic features with sRNAs discovered in animals [7,8].

Many breakthroughs have been made in identifying different classes of sRNAs of various origins, as well as elucidating their biogenesis pathways and functions in diverse plants species. Initially, several groups adopted cloning methods to identify plant sRNAs [7,9,10,11]. This approach is labor-intensive and time-consuming. The growth of high-throughput next-generation sequencing (NGS) has accelerated the discovery of novel plant sRNAs by transcriptome-wide investigations of sRNA profiles. Numerous bioinformatics tools were developed to analyze the sequencing data, identify sRNAs of interest and predict their targets. Successful predictions of sRNA targets have helped researchers to further investigate the mechanisms of sRNA regulations in plants. Moreover, a series of experimental strategies have been invented, optimized, and modified to overcome the challenging tasks in validating sRNA expressions and functions. In this review, the current understanding on sRNAs in abiotic stress, the strategies to identify these sRNAs, and the functional validations of sRNAs will be discussed.

## 2. Mechanisms of sRNA-Mediated Genetic Regulation

### 2.1. Transcriptional Gene Silencing

**Figure 2:**
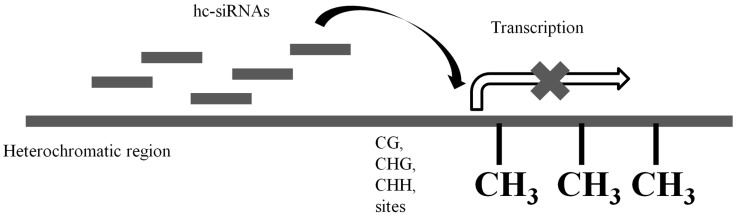
hc-siRNAs are transcribed at the heterochromatic regions where they act in *cis* to trigger the methylation of cytosine in these sequence contexts: CG, CHG and CHH [17,18,19], resulting in transcriptional silencing.

sRNA-directed DNA methylation leads to the inhibition of transcription [12]. This phenomenon is known as transcriptional gene silencing (TGS). TGS is also termed homology-dependent gene silencing (HDGS) as it requires the sequence homology between the sRNA and the promoter [13,14]. Recent studies have revealed the roles of sRNAs in DNA methylation. It was demonstrated in tobacco that double-stranded RNA-(dsRNA-)triggered TGS and promoter methylation involved the production of sRNA (~23 nt) [14]. siRNAs of heterochromatic origin (hc-siRNA) have recently been identified as a functionally distinct subset of siRNAs which are involved in inducing RNA-directed DNA methylation (RdDM) [15,16]. These 24 nt hc-siRNAs are transcribed at the heterochromatic regions where they trigger the methylation of cytosine, in these sequence contexts: CG, CHG and CHH, in *cis* [17,18,19]. Figure 2 shows the role of hc-siRNAs in TGS. Besides typical sRNA biogenesis components (RDR, DCL, AGO), the mechanism of *de novo* hc-siRNA-induced RdDM involves both RNA polymerases (Pol) IV and V, which probably transcribe the double-stranded precursors and facilitate methylation at the target sites respectively [15,20].

There are extensive studies showing the relationships between DNA methylation and abiotic stresses [21]. It was shown that the decrease in the salt tolerance capability of *Arabidopsis* coincided with the blocking of DNA methylation at cytosine by 5-azacytidine treatment [22]. It was also found that there is a positive relationship between the dosage of irradiation and the extent of global genome methylation in pine trees [23]. There are some studies showing the association between sRNAs and DNA methylation under abiotic stress. For example, in *Prunus persica*, a number of cold-responsive microRNAs (miRNAs) were predicted to target genes involved in DNA methylation [24].

### 2.2. Post-Transcriptional Gene Silencing

**Figure 3:**
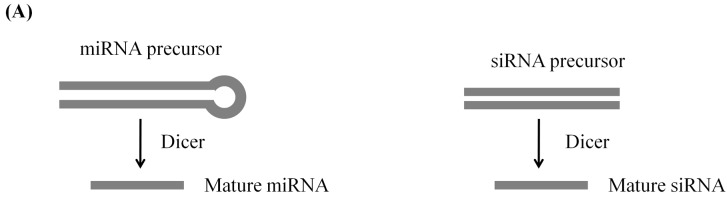

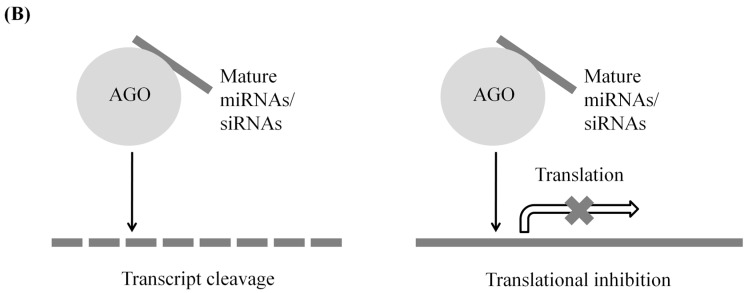
The roles of miRNA and siRNA in PTGS (post-transcriptional gene silencing). (**A**) The precursor of miRNA is a self-complementary RNA which forms a hair-pin structure while the precursor of siRNA is a dsRNA. The precursors are diced to form mature miRNA or siRNA [7,28,29]; (**B**) The mature miRNA or siRNA interacts with the AGO (argonaute) protein to form RISC (RNA-induced silencing complexes), which causes the silencing of the target gene by transcript cleavage or translational inhibition [7,28,29].

Based on the differences in their biogenesis pathway, sRNAs in plants are usually classified into two major groups: miRNAs and endogenous siRNAs. miRNAs and siRNAs are derived from structurally different precursors (Figure 3). The precursors of miRNAs are near-perfectly or perfectly self-complementary, forming a hair-pin-like loop. On the contrary, siRNA precursors are double-stranded, extensively complementary. In spite of the structural differences in their precursors, the biogenesis pathways of siRNAs and miRNAs essentially resemble each other [25,26]. The early precursors of sRNAs are usually transcribed by RNA polymerases (Pol) II, IV and V [27]. The miRNA precursors fold to form hair-pins, while siRNA precursors are converted to dsRNAs by RNA-dependent RNA polymerases (RDR). Precursors are then diced into mature miRNAs or siRNAs by dicer-like (DCL) proteins [7,28,29]. Mature miRNAs and siRNAs are single-stranded and can interact with argonaute (AGO) proteins to form RNA-induced silencing complexes (RISC). Depending on the type of AGO involved, PTGS could silence targets through cleavage of the transcript or translational inhibition by RISC [30,31]. The biogenesis pathways of miRNAs and siRNAs use distinct sets of Pol, RDR, DCL and AGO. This highlights the differences between the two major classes of sRNAs [26]. sRNA-mediated gene regulation mechanisms are summarized in Table 1.

**Table 1:** Summary of sRNA-mediated gene regulation mechanisms.

Mechanism of Regulation	sRNA Types Participated	Origin of sRNAs	Targets of sRNAs	Modes of Action
Transcriptional gene silencing	hc-siRNAs	Transcripts of heterochromatic regions	Heterochromatic regions (act in *cis*)	RNA-directed DNA methylation
Post-transcriptional gene silencing	miRNAs	Short stem-loop-forming transcripts	Other transcripts (act in both *cis* and *trans*)	Transcript cleavage; translational inhibition
			*TAS-*transcripts	Triggering double strand synthesis of *TAS*-transcripts
	NAT-siRNAs	Antisense transcripts	Other transcripts in both *cis* and *trans*	Transcript cleavage; translational inhibition
	ta-siRNAs	*TAS*-loci derived transcripts	Other transcripts in both *cis* and *trans*	Transcript cleavage; translational inhibition

*TAS*-transcripts, ta-siRNA transcripts; TAS-loci, ta-siRNA generating loci.

#### 2.2.1. miRNA

Most efforts have been devoted to an identification of conserved and lineage-specific miRNAs. Currently, it is known that many plant miRNA target genes are involved in developmental processes, and the disruption of miRNA biogenesis generally results in developmental abnormalities such as the accelerated growth of lateral roots in the early seedling stage [32]. The cleavage of mRNAs directed by miRNAs was demonstrated in *Arabidopsis* [33]. The advancement of high-throughput sequencing has facilitated the identification of abiotic stress-responsive miRNAs. For example, it was reported that the expression level of miR398 was decreased after Cu^+^, Fe^+^, ozone, and salt treatments [34,35]. The identification of the cleavage targets of miR398 has enabled the understanding of the functions of this miRNA under abiotic stresses [36].

#### 2.2.2. siRNA

Plant siRNAs are more diverse than miRNAs in terms of sizes, structures of precursors, genomic origins and functions. *Trans*-acting siRNAs (ta-siRNAs), natural antisense transcript-derived siRNAs (NAT-siRNAs) and the newly identified hc-siRNAs are relatively well-studied subsets. The role of hc-siRNAs has been discussed above in Section 2.1. While many other siRNAs are involved in PTGS, the newly classified hc-siRNA is involved in TGS [17,18,19,37,38,39,40].

Ta-siRNAs are derived from ta-siRNA generating loci (TAS loci) [37,38]. The existence of ta-siRNAs revealed that sRNA regulations in plants form complex and multi-level networks through miRNA-induced signal transduction [41]. A miRNA-guided cleavage of TAS transcripts triggers the conversion of these transcripts to double-stranded ta-siRNA precursors, which can be diced in-phase to produce 21–22 nt ta-siRNAs [42]. Hence, a number of ta-siRNAs can arise along the ta-siRNA transcripts (TAS transcripts) neither with gaps nor overlaps. This phasing feature has been frequently used to predict novel ta-siRNA generating gene (*TAS* genes) and hence some researchers also refer ta-siRNAs as phased siRNAs (phasiRNAs) [43,44]. Four *TAS* gene families have been discovered in *Arabidopsis thaliana* [45,46,47]. TAS3 is well known for its function in determining the developmental timing and pattern by targeting to the *AUXIN RESPONSE FACTOR 3* [48]. Hypoxia-responsive ta-siRNAs from the TAS1, TAS2 and TAS3 families have also been reported [49].

NAT-siRNAs refer to the natural antisense transcript-(NAT-) derived siRNAs. The double-stranded precursors are generated at the overlapping region of 2 partial or perfectly complementary transcripts [39,50]. Deep sequencing analyses on *Arabidopsis thaliana* suggested the occurrence of 2 classes of NAT-siRNAs: (i) 20–22 nt, DCL-1-dependent; and (ii) 23–28 nt, DCL-3-dependent NAT-siRNAs [51]. NAT-siRNAs can be further classified based on the origins of their NAT precursors, with *cis*-NATs being derived from the same loci [39,40,52] and *trans*-NATs from remote loci of their sense counterparts [40,53]. Genome-wide searches for overlapping short transcripts were successfully applied in *Oryza sativa* [53] and *Petunia hybrida* [54] to predict NAT-siRNAs. Through deep sequencing, NAT-siRNAs responsive to abiotic stresses including salt, cold, and drought have been reported [55].

## 3. Computational Methods to Identify sRNAs

As discussed above, in most of the recent research, abiotic stress-responsive sRNAs are identified by comparing the sRNA profiles between treated and untreated plants through high-throughput sequencing. Therefore, reliable computational tools are crucial for the identification of sRNAs involved in the gene regulation network.

### 3.1. Computational Prediction of miRNA Gene Loci

Most of the mature plant miRNAs are 21–24 nt long. From the analyses of eight plant species, 84% of plant miRNA loci are found in clusters at intergenic regions, with a few exceptions found in intronic regions [56]. In contrast to animal miRNAs which are frequently clustered together, only ~20% of plant miRNA genes are clustered together, and these clustered genes often encode miRNAs belonging to the same family or targeting genes of the same protein family [57]. The usual characteristic actions of miRNAs in plants in *trans* and the hairpin structure of their precursors are hallmarks frequently employed by common miRNA prediction tools. Given the well-developed and relatively standard prediction pipeline, an overwhelming number of miRNAs has been documented in different plant species. These classical prediction tools are summarized in Table 2.

**Table 2:** Summary of classical miRNA prediction tools.

Tool	Application	Property	Reference
MIRFINDER	Detection of potential conserved miRNAs in *Arabidopsis thaliana* and *Oryza satica*	The use of NCBI BLAST to search for conserved short hits (~21–22 nt). The hits with flanking sequences were identified as putative hairpin precursors.	[58]
miRSeeker	Identification of novel miRNA candidates that are conserved in insect, nematode, or vertebrate	The use of AVID to align *Drosophila melanogaster* and *Drosophila pseudoobscura* euchromatic sequences to search for conserved sequences meeting these two criteria: 1. Having extended stem-loop structure; 2. Having nucleotide divergence from known miRNAs.	[59]
mirCoS	Prediction of mammalian miRNAs	Detection of known miRNAs and prediction of new miRNAs based on sequence, secondary structure and conservation by comparing human and mouse genomes.	[60]
miRRim	Identification of novel miRNAs in human	Detection of miRNAs with the use of a hidden Markov model.	[61]
miRAlign	Detection of miRNA homologs or orthologs in animals.	Detection of miRNAs based on sequence and structure alignment. The sensitivity is better than BLAST search and ERPIN search with comparable specificity.	[62]
microHARVESTER	Identification of plant miRNA homologs	Identification of plant miRNA homologs based on query miRNA.	[63]
MiRscan	Identification of vertebrate miRNA genes	Evaluation of conserved stem-loops.	[64]
miRDeep	Identification of miRNAs with deep sequencing data	The use of known miRNA training set obtained from *Caenorhabditis elegans* to deduce parameters of most probable miRNA precursors. These parameters were used to score precursor candidates using a probabilistic approach.	[65]
MiRCheck	Identification of miRNAs in *Arabidopsis thaliana* and *Oryza sativa*	The use of EINVERTED from EMBOSS [66] to predict genome-wide inverted repeats in both *Arabidopsis thaliana* and *Oryza sativa* to define possible hairpin regions, and the check for segments with high homology between *Arabidopsis thaliana* hairpins and *Oryza sativa* hairpins using Patscan.	[67]

NCBI, National Center for Biotechnology Information; BLAST, Basic Local Alignment Search Tool; AVID, a global alignment program; ERPIN, Easy RNA Profile IdentificatioN; EINVERTED, a program that finds inverted repeats in nucleotide sequences; EMBOSS, European Molecular Biology Open Software Suite.

#### 3.1.1. Choosing the Right Tools for Plant miRNA Discovery

There are some key differences between animal and plant miRNAs [68]. For example, the plant stem-loop precursors are more variable in length. Longer precursors usually have smaller minimum free energy (MFE) and hence will increase the false-positive rate of miRNA prediction [69]. Therefore, some programs such as miRDeep-P [70] and a newer graphical user interface (GUI) application, miRPlant [71], have set a maximal value for the log-odds score for the MFE metric based on the Gumbel distribution to model the distribution of MFE.

The length of plant miRNA precursors ranged from 50 to 930 nt with a mean length of 146 nt; whereas animal precursors have a range of 45 to 215 nt and a mean length of 87 nt [69]. The program miRPlant includes both short and long plant miRNA precursors to solve the issue of length variability, by scanning the peak expressed region. Both 100 and 200 nt sliding windows are scanned when predicting the secondary structure of the precursors [71]. The program MIReNA is also plant miRNA-detection-friendly. In this method, MFE metric is normalized by the length and then GC content of the precursors to obtain the final minimum free energy index (MFEI). Using this approach, rice miRNAs were successfully discovered [72].

**Figure 4:**
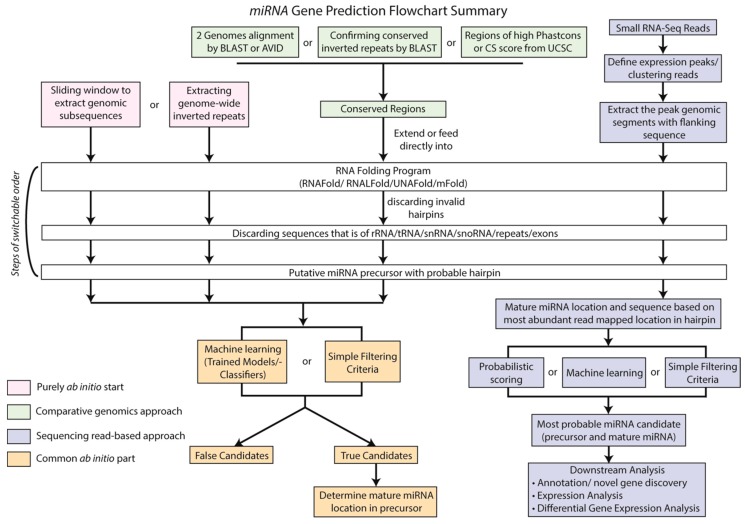
A flowchart for miRNA gene prediction. This flowchart summarized how computational tools predict miRNAs with different approaches. Purely *ab initio* miRNA prediction programs (pink boxes) use the reference genome of interest as the only starting material to generate miRNA precursor candidates, followed by classifying/filtering with known miRNA properties. In contrast, comparative genomics miRNA prediction programs (green boxes) start with identifying conserved regions between two or more genomes to generate miRNA precursor candidates, followed by the same classifying/filtering step of purely *ab initio* prediction programs (orange boxes). The sequencing read-based prediction programs (purple boxes) use miRNA expression data to locate possible mature miRNAs. Subsequently, flanking genomic regions of mapped reads are extracted and evaluated whether they pass the criteria of miRNA annotation, using various scoring/classifying algorithms.

Another difference between plant and animal miRNAs is the degree of conservation in certain segments of the miRNA precursors. For instance, the miRDeep core algorithm, designed for animals [73], scores the sequence conservation in the proposed seed region [73], which is the 2nd–7th nucleotides of the predicted mature miRNA. In contrast, plant miRNA mature sequences are conserved in two positional blocks, from the 2nd to 13th nucleotide and from the 16th to the 19th nucleotide, with the 4th nucleotide strictly conserved [69]. Therefore, it is important to use plant-friendly tools for plant miRNA identification. These plant-friendly tools are summarized in Table 3. Figure 4 shows the workflow of miRNA gene prediction.

**Table 3:** Summary of plant-friendly miRNA prediction tools using deep sequencing data.

Tool	Property	Reference
miRDeep-P	Adopting miRDeep core algorithm with modified step of setting a maximal value for the MFE log-odds score to account for longer plant miRNA precursors	[70]
miRPlant	Implementing miRDeep* [74] with 100 and 200 nt extended genomic regions from mapped read peaks to include more bona fide miRNA precursor candidates	[71]
miR-PREFeR	Filtering miRNA precursor candidates with criteria suggested in [75] for annotating plant miRNAs	[76]
MIReNA	Filtering putative precursors with length-normalized and GC-normalized MFE to accommodate the prediction of plant miRNAs	[72]
ShortStack	Defining structural miRNA parameters based on selected annotated miRNA in miRBase depending on the “miRType” specified by user, either “plant” or “animal”, subsequently filter candidates with criteria suggested in [75]	[77]

#### 3.1.2. Computational Prediction of TAS-Like Loci

Based on the characteristics of ta-siRNAs, computational methods have been employed to predict TAS-like loci in plants. Since the phased 21 nt increments have been observed for all known TAS loci in *Arabidopsis thaliana*, the algorithm utilizes the phasing feature to predict novel TAS loci [78]. Usually 11 cycles of a 231-bp window of sequence downstream of the 5′ mapping start site of each sRNA are examined. Significant occurrences from random events can be identified by calculating the *p*-value for *k*-phased sRNAs, and a *p*-value between 0.0005 and 0.001 is considered stringent. The following equation shows the *p*-value calculation:

(1) $$Pr\left(X=k\right)=\;\frac{\left(\begin{array}{c} 440\\ n-k \end{array}\right)\left(\begin{array}{c} 21\\ k \end{array}\right)}{\left(\begin{array}{c} 461\\ n \end{array}\right)}$$

where *k* is the number of distinct sRNAs mapped to phased positions and n is the total distinct sRNAs observed in a 231-bp fragment.

(2) $$p\left(k\right)=\overset{21}{\underset{X=k}{\sum }}\mathrm{Pr}\;\left(X\right)$$

The algorithm can also be modified to predict phasiRNAs with phasing intervals other than 21 nt, such as that used by the plant sRNA regulatory cascade analysis server pssRNAMiner [79]. In this revised version, the number of positions having sRNAs are mapped, rather than counting the number of distinct sRNAs [79], with a new variable (±2 nt) added to reflect the offset [47] of the cleavage positions of phased sRNAs to phased positions.

Another method to evaluate phased regions is to use the phase score (*P*) [80] to examine each of the 8-cycle windows using 454 pyrosequencing reads, and its calculation is shown below:

(3) $$P=\text{In }\left[{\left(1+\;\overset{8}{\underset{i=1}{\sum }}k_{i}\right)}^{n-2}\right],\;P>0$$

where *n* is the number of phased cycle positions occupied by at least one sRNA read and *k* is the total number of reads with consolidated start coordinates in a given phase. An adjusted version of phase score was proposed [81] which was fitted to analyze the Illumina sequencing reads.

#### 3.1.3. Common Features of Target Prediction Tools

There are a few considerations in the design and development of sRNA target prediction tools. First of all, sRNAs are mapped to mRNA transcripts to get a set of potential targets. BLAST [82] and Smith-Waterman algorithm [83] are widely used in sequence alignments. Based on the phenomenon that sRNAs are highly complementary to their mRNA targets, the sequence similarity and binding pattern in potential sRNA-target pairs are further modeled into scoring schemes [84,85,86,87,88,89,90,91]. The number of mismatches, insertions/deletions (indels) and gaps allowed in miRNA-mRNA alignment are limited in sequence similarity requirements, and mismatches/indels/gaps at positions near the 5′ ends of miRNAs are further punished. Besides examining the complementarity between sRNA and target sequences, psRNATarget [92] also integrates reverse complementary matching and target-site accessibility, which in turn is evaluated by the energy required to “open” secondary structures around target sites on mRNAs.

#### 3.1.4. Functions of Prediction Tools

Most prediction methods/tools are used to search for potential targets for query sRNAs [45,84,85,87,88,89,92], and some search potential sRNAs that can target to the query mRNAs [86]. In addition to reporting target mRNAs, psRNATarget also predicts if the sRNA regulatory effect is at the post-transcriptional or translational level. The sRNA regulatory effect is reported as translational inhibition when a mismatch is detected in the central complementary region of the sRNA sequence [92].

### 3.2. Computational Prediction of sRNA Targets

Plant RNA targets have been computationally predicted on the basis of their extensive complementarity to the miRNAs. A variety of computational tools specific for plant miRNAs have been developed in recent years, including miRU [85], C-mii [87], TAPIR [91], miRTour [93], psRNATarget [92], and Targetfinder [45,84], as opposed to tools designed for mammalian miRNAs such as miRanda [94] and RNAHybrid [95]. Several principles of plant miRNA target prediction include an alignment scoring scheme, target site accessibility [96,97] and mapping patterns between miRNAs and mRNA targets (such as G:U wobbles and mismatches), which are commonly applied in many tools and methods [45,84,85,86,87,89,90,91,93].

Ta-siRNAs interact with target RNAs by the same mechanism as miRNAs [45], and thus the current prediction methods for ta-siRNA targets are similar to those of miRNAs. psRNATarget can detect targets for both miRNAs and ta-siRNAs using the same strategy. In a study on grapevine [89], ta-siRNA targets were predicted by applying standard BLASTn [82] without any filters to search for complementarity between ta-siRNA and transcripts. The results of target prediction based on sequence similarity using BLASTn alone need further validation to reduce false-positive hits that show high similarity to the real targets.

### 3.3. High-Throughput sRNA Target Identification—Degradome

Given the high false-positive rate, high-throughput experimental confirmation of computational predicted targets is an important step to expedite sRNA researches. Degradome sequencing [98], also known as parallel analysis of RNA ends (PARE) [99], provides a high-throughput strategy for the global experimental identification of targets for miRNAs, ta-siRNAs and NAT-siRNAs. The protocol for constructing a degradome library is modified from RLM-RACE, in order to sequence millions of 5′ uncapped ends of RNA fragments originating from poly-A RNAs [99,100]. Some of these fragments represent cleavage signatures as a result of sRNA regulation. To identify miRNA targets, degradome sequencing reads are first mapped to genome/transcripts, then these reads are extended a few nucleotides both upstream and downstream to retrieve the extended degradome tags. If the 10th position of a miRNA was aligned to the starting position of a read on an extended degradome tag, the tag will be reported as the miRNA cleavage signature [98,101]. To identify a true miRNA cleavage from background noise, target plots (t-plots) (a function of degradome reads abundance against the position on a transcript) can be used. The true miRNA cleavages usually have a high abundance at a specific position on a t-plot. An automated plant-compatible pipeline CleaveLand was developed to facilitate the interpretation of degradome data [102]. For ta-siRNA and NAT-siRNA targets, similar and simpler methods are employed. SiRNAs of lengths 20–22 nt were aligned to degradome tags, and alignments indicated cleavage of mRNA [102]. Degradome analyses can significantly increase the precision of sRNA target identification and reduce the number of false-positive targets. However, its major drawback is the requirement of a large amount of RNA inputs for the library preparation [99,100].

## 4. Experimental Validations of Predicted sRNAs

The genome-wide identification of sRNAs by deep RNA sequencing has been a popular strategy to search for abiotic stress-responsive sRNAs. However, deep sequencing has to be followed by experimental validation to provide the biological context for the big data set. The existence of the predicted sRNAs, targets of the sRNAs, and the biological functions of the sRNAs have to be validated. The classical validation methodologies are summarized in Table 4.

**Table 4:** Summary of experimental methodologies previously used for sRNA studies.

Method	Stress	sRNA	Reference
**Validation of the existence of sRNA**			
qRT-PCR	Salinity, copper deficiency	miR397, miR857	[103]
Northern blot	Salinity, sulphur deprivation, oxidative stress, nitrogen deficiency, inorganic phosphtase deprivation, drought, irradiation, copper deficiency	miR399, miR395, miR398, miR408	[34,90,104,105,106,107,108,109,110]
**Validation of the target gene**			
5′ RACE	Copper deficiency	miR397, miR408	[103]
**Transgenic plant for functional test**			
*Arabidopsis*	Inorganic phosphate deprivation	miR399	[104]
*Arabidopsis*	Drought	miR196	[111]
Creeping bentgrass	Drought, salinity	miR319	[112]

Classical validation methodologies suffer from various shortcomings. Therefore, we will discuss below the recent advancements and improvements of these validation methodologies.

### 4.1. Validation of sRNAs Expression

#### 4.1.1. Quantitative Detection of sRNAs by Northern Blot

Northern blot is a traditional method for studying gene expression. However, this method suffers from low sensitivity and low throughput. Despite these drawbacks, northern blot is still a valuable tool for studying the sRNA size, differentiating sRNAs of highly similar sequences, and detecting a mature sRNA and its precursor simultaneously [113]. These northern blots have been modified specifically for detecting sRNAs by improving probe synthesis and cross-linking methods. Traditional cross-linking methods for mRNA, such as heat or alkaline-assisted fixation, are not suitable for sRNAs due to their small sizes. On the other hand, UV-mediated cross-linking can potentially reduce the detection sensitivity [113]. Locked nucleic acid (LNA), an RNA analog containing a modified ribose moiety, has been used to modify oligonucleotide probes to improve detection sensitivity [114]. In principle, LNAs are incorporated into every 3rd nucleotide position on LNA-modified probes, resulting in better binding specificity and sensitivity than traditional DNA probes [114]. A chemical cross-linking method using 1-ethyl-3-(3-dimethylaminopropyl) carbodiimide (EDC) has also been introduced to improve detection sensitivity [113]. EDC cross-linking facilitates the formation of covalent bonds between the terminal phosphate of RNAs to the amino group on the nylon membrane, and the detection sensitivity can be enhanced by up to 50 folds [113]. Based on LNA-modified probes (L), EDC cross-linking (E) and traditional digoxigenin (DIG)-labeled probes (D), an LED protocol was further developed to enhance the detection sensitivity for rare sRNAs [115]. However, due to the short lengths of sRNA, there is a high possibility that different sRNAs may share similar sequences.

#### 4.1.2. Quantitative Detection of sRNAs by qPCR

An obvious advantage of qPCR is that the amount of starting RNA material is significantly lower than that required for a northern blot [116]. Unlike traditional mRNA expression studies by qPCR, the length of sRNAs is too short to allow the annealing of PCR primers at both ends. There are mainly two types of detection methods used in qPCR, namely interchelating dye-based and probe-based methods [117]. For interchelating dye-based detection, the experimental procedures include the isolation of sRNAs, addition of poly-A at the 3′ ends by polyadenylation and reverse transcription using a primer consisting of poly-T at the 3′ end and adaptor sequence at the 5′ end, followed by qPCR using an sRNA-specific primer and an adaptor-specific primer [116]. For the probe-based detection method, TaqMan PCR is a common strategy [118]. Unlike the interchelating dye-based method, TaqMan PCR does not involve polyadenylation. Reverse transcription is mediated by a stem-loop primer which is complementary to the sRNA at its 3′ end with an adaptor sequence at its 5′ end. Using an sRNA-specific primer and an adaptor-specific primer, PCR is performed in the presence of a TaqMan probe for detection [118]. To improve the sensitivity of sRNA-qPCR, Zip Nucleic Acids (ZNAs) can be used as qPCR primers by their rapid binding to target DNAs and stably enhance the amplification of rare DNA species [119,120]. Common strategies of normalization involve the use of endogenous house-keeping genes or the external spike-in of control oligonucleotides that relies on the accuracy of quantifying the oligonucleotide used [117].

#### 4.1.3. *In Situ* Hybridization for Spatiotemporal Detection of sRNAs

*In situ* hybridization allows the tissue-specific and spatiotemporal detection of sRNAs [121]. DIG-labeled probes are commonly used in *in situ* hybridization. However, its sensitivity and specificity of detection have been challenged [121]. Recently, a refined protocol using LNA probes was introduced in a number of plant tissues [121]. In this protocol, the permeability of sample tissue is improved by protease treatment to enhance detection sensitivity. Triethanolamine-acetic anhydride (TEA) is also used to treat the sample tissue to reduce non-specific binding between the probe and positively-charged amino acids, and RNase is used in the post-hybridization wash to reduce non-specific binding [121]. As a low-cost alternative, ZNA probes for whole-mount *in situ* hybridization have been successfully used in *Arabidopsis* tissues [122].

### 4.2. Validation of sRNA Targets

The functional validation of sRNAs involves the validation of binding targets predicted computationally and the study of the functional roles of the sRNAs of interest in the biological systems.

#### 4.2.1. Labeled miRNA Pull-down (LAMP) Assay System

The LAMP assay system was employed successfully in zebrafish and *C. elegans* as an experimental approach to validate the target transcripts of a miRNA of interest [123]. In the LAMP assay system, the pre-miRNA is DIG-labeled and mixed with a cell extract to allow the *in vitro* production of mature miRNA and the binding between the miRNA with its target [123]. The miRNA-transcript complex is then pulled down by immunoprecipitation [123]. This approach is relatively straightforward. However, it has been criticized that the presence of the DIG group may influence the processing of pre-miRNA as well as the binding of the miRNA to its target [124]. Furthermore, the specificity of this *in vitro* approach is also questionable [124]. Hence, the LAMP assay is not yet popular in the study of plant systems.

#### 4.2.2. RNA Ligase-Mediated Amplification of cDNA End (RLM-RACE)

RLM-RACE involves the isolation of mRNAs with a poly-A tail, followed by the ligation of an RNA adaptor at the 5′ end of the cleavage product which contains the 5′ monophosphate available for ligation [33]. After reverse transcription using an oligo-T (dT) primer, the cDNAs of the cleavage products are amplified with the use of a 5′ adaptor-specific primer and a gene-specific primer. The PCR product is then confirmed by sequencing [33]. Since RLM-RACE yields the cDNAs of all cleaved mRNAs with 5′ monophosphate available for ligation, this method cannot distinguish which type of sRNAs are involved in mRNA cleavages.

### 4.3. Functional Validation of sRNAs

To have better understanding of the biological roles of the sRNAs, functional analyses are necessary. Functional analyses include the study on the regulatory roles, either positive or negative, of sRNAs and also the validation of predicted sRNA targets. The validation of the predicted sRNA targets usually involves reporter assays, while the study on sRNA regulatory roles includes gain-of-function or loss-of-function approaches.

#### 4.3.1. Reporter Assays

Reporter assays are *in vivo* approaches to validate the binding between the sRNA of interest and the predicted targets. These assays involve the transient or stable expression of a construct composed of the target sequence of sRNA at the 3′ UTR downstream to a reporter gene such as green fluorescence protein (GFP) and luciferase. When the sRNA of interest and the reporter gene are co-expressed, the transcripts of the reporter gene will be cleaved, leading to a reduction of the reporter signal [125]. However, such reporter assays involve transgenesis which could be a limiting factor for some plant species.

#### 4.3.2. Validation of the Effect of the sRNA of Interest on the Target Gene Expression

The effect of sRNAs can be either on the transcript cleavage or the inhibition of translation [126]. Therefore, it is expected that the change in sRNA expressions would alter the expression of target genes at the transcript level or protein level. Over-expression and knock-down of the sRNA concerned are common strategies to investigate the effects of the sRNA abundance on the target transcript expression [125]. The knock-down of sRNA is commonly done by transforming the anti-sense sRNA into the system to be studied [125]. Following the study of the effects of the sRNA on the target gene expression, physiological investigations are needed to understand the response of the biological system to the altered sRNA expression level. Similar to reporter assays, such over-expression and knock-down approaches are challenging in many plant systems that are not readily transformable. T-DNA insertion has been a common approach in inducing plant mutagenesis [127]. However, the efficiency of sRNA precursor mutations by T-DNA insertion is not satisfactory [127,128]. In the genome-wide mutagenesis study in *Arabidopsis thaliana*, over 225,000 T-DNA insertion events resulted in the identification of only 21,799 mutations of genes [127]. Thus alternative approaches have been developed to validate the function of the sRNA of interest. Using *Arabidopsis* as a model, the artificial miRNA (amiRNA) technology was introduced to silence endogenous miRNA [128]. The amiRNA technology works with *Agrobacterium*-mediated transformation of *Arabidopsis thaliana* to introduce the amiRNA into the genome [128]. If an amiRNA is designed to target the mature miRNA, it will result in the silencing of the whole family of the miRNA [128]. On the contrary, if the amiRNA is designed to target the stem-loop of the miRNA precursor, it will silence a specific targeted miRNA family member [128]. This method has been demonstrated in the sRNA-guided cleavage of pre-sRNAs in the plant nucleus so far [128]. Other than modulating the expression of the sRNA concerned, the plant can also be transformed with the modified target of the sRNA in the target mimicry approach where the function of the sRNA will be negated [129]. A comparison of sRNA validation methods is shown in Table 5.

**Table 5:** Comparison of methods for sRNA validation.

Purpose	Method	Advantage(s)	Disadvantage(s)
Validation of the existence of predicted sRNA	Northern blot	Quantitative, simultaneous detection of sRNA and its precursor	Optimization steps are needed to improve sensitivity and specificity.
	qPCR	Small amount of RNA is required	Normalization by spike-in control or housekeeping genes can be unreliable.
Validation of the existence of predicted sRNA	*In situ* hybridization	Allows tissue-specific and spatiotemporal detection	Optimization steps are needed to improve sensitivity and specificity.
Functional analysis of sRNA	LAMP assay	Straightforward	An *in vitro* approach, the pre-miRNA processing and specificity have been questioned; not popular for plants.
	RLM-RACE	Previous knowledge of the cleaved mRNA is not required	Cannot distinguish by which type of sRNA the mRNA cleavage is mediated.
	Reporter assays	An *in vivo* approach	Transformation of the species under study is needed.

## 5. Conclusions

Different classes of sRNAs have been shown to associate with abiotic stresses. Computational prediction is a powerful approach to perform a genome-wide search of sRNAs. However, special attention should be paid to plant sRNA analyses since the methods used in animal research need to be optimized for their proper use in plant applications. Moreover, experimental validation is an essential step before making conclusions on the biological functions of the identified sRNAs.

## References

[1] Nakashima K., Yamaguchi-Shinozaki K., Shinozaki K. (2014). The transcriptional regulatory network in the drought response and its crosstalk in abiotic stress responses including drought, cold, and heat. Front. Plant Sci..

[2] Gehan M.A., Greenham K., Mockler T.C., McClung C.R. (2015). Transcriptional networks—Crops, clocks, and abiotic stress. Curr. Opin. Plant Biol..

[3] Priest H.D., Fox S.E., Rowley E.R., Murray J.R., Michael T.P., Mockler T.C. (2014). Analysis of global gene expression in *Brachypodium distachyon* reveals extensive network plasticity in response to abiotic stress. PLoS ONE.

[4] Hamilton A.J., Baulcombe D.C. (1999). A species of small antisense RNA in posttranscriptional gene silencing in plants. Science.

[5] Dalmay T., Hamilton A., Mueller E., Baulcombe D.C. (2000). *Potato virus X* amplicons in *Arabidopsis* mediate genetic and epigenetic gene silencing. Plant Cell.

[6] Hutvagner G., Mlynarova L., Nap J.P. (2000). Detailed characterization of the posttranscriptional gene-silencing-related small RNA in a *GUS* gene-silenced tobacco. RNA.

[7] Reinhart B.J., Weinstein E.G., Rhoades M.W., Bartel B., Bartel D.P. (2002). MicroRNAs in plants. Genes Dev..

[8] Bartel D.P. (2004). MicroRNAs: Genomics, biogenesis, mechanism, and function. Cell.

[9] Llave C., Kasschau K.D., Rector M.A., Carrington J.C. (2002). Endogenous and silencing-associated small RNAs in plants. Plant Cell.

[10] Sunkar R., Zhu J.K. (2004). Novel and stress-regulated microRNAs and other small RNAs from *Arabidopsis*. Plant Cell.

[11] Sunkar R., Girke T., Jain P.K., Zhu J.K. (2005). Cloning and characterization of microRNAs from rice. Plant Cell.

[12] Baulcombe D. (2004). RNA silencing in plants. Nature.

[13] Kooter J.M., Matzke M.A., Meyer P. (1999). Listening to the silent genes: Transgene silencing, gene regulation and pathogen control. Trends Plant Sci..

[14] Mette M., Aufsatz W., van der Winden J., Matzke M., Matzke A. (2000). Transcriptional silencing and promoter methylation triggered by double-stranded RNA. EMBO J..

[15] Matzke M., Kanno T., Daxinger L., Huettel B., Matzke A.J. (2009). RNA-mediated chromatin-based silencing in plants. Curr. Opin. Cell Biol..

[16] Creasey K.M., Zhai J., Borges F., van Ex F., Regulski M., Meyers B.C., Martienssen R.A. (2014). miRNAs trigger widespread epigenetically activated siRNAs from transposons in *Arabidopsis*. Nature.

[17] Hamilton A., Voinnet O., Chappell L., Baulcombe D. (2002). Two classes of short interfering RNA in RNA silencing. EMBO J..

[18] Xie Z., Johansen L.K., Gustafson A.M., Kasschau K.D., Lellis A.D., Zilberman D., Jacobsen S.E., Carrington J.C. (2004). Genetic and functional diversification of small RNA pathways in plants. PLoS Biol..

[19] Zilberman D., Cao X., Jacobsen S.E. (2003). Argonaute4 control of locus-specific siRNA accumulation and DNA and histone methylation. Science.

[20] Law J.A., Jacobsen S.E. (2010). Establishing, maintaining and modifying DNA methylation patterns in plants and animals. Nat. Rev. Genet..

[21] Khan A., Shah S., Irshad M. (2015). Immediate and transgenerational regulation of plant stress response through DNA methylation. J. Agric. Sci..

[22] Boyko A., Blevins T., Yao Y., Golubov A., Bilichak A., Ilnytskyy Y., Hollander J., Meins F., Kovalchuk I. (2010). Transgenerational adaptation of *Arabidopsis* to stress requires DNA methylation and the function of dicer-like proteins. PLoS ONE.

[23] Kovalchuk O., Burke P., Arkhipov A., Kuchma N., James S.J., Kovalchuk I., Pogribny I. (2003). Genome hypermethylation in *Pinus silvestris* of chernobyl—A mechanism for radiation adaptation?. Mutat. Res. Fundam. Mol. Mech. Mutagen..

[24] Barakat A., Sriram A., Park J., Zhebentyayeva T., Main D., Abbott A. (2012). Genome wide identification of chilling responsive microRNAs in *Prunus persica*. BMC Genom..

[25] Meister G. (2013). Argonaute proteins: Functional insights and emerging roles. Nat. Rev. Genet..

[26] Carthew R.W., Sontheimer E.J. (2009). Origins and mechanisms of miRNAs and siRNAs. Cell.

[27] Zheng B., Wang Z., Li S., Yu B., Liu J.-Y., Chen X. (2009). Intergenic transcription by RNA polymerase II coordinates Pol IV and Pol V in siRNA-directed transcriptional gene silencing in *Arabidopsis*. Genes Dev..

[28] Dunoyer P., Himber C., Voinnet O. (2005). Dicer-like 4 is required for RNA interference and produces the 21-nucleotide small interfering RNA component of the plant cell-to-cell silencing signal. Nat. Genet..

[29] Gasciolli V., Mallory A.C., Bartel D.P., Vaucheret H. (2005). Partially redundant functions of *Arabidopsis* dicer-like enzymes and a role for DCL4 in producing *trans*-acting siRNAs. Curr. Biol..

[30] Baumberger N., Baulcombe D.C. (2005). *Arabidopsis* argonaute1 is an RNA slicer that selectively recruits microRNAs and short interfering RNAs. Proc. Natl. Acad. Sci. USA.

[31] Takeda A., Iwasaki S., Watanabe T., Utsumi M., Watanabe Y. (2008). The mechanism selecting the guide strand from small RNA duplexes is different among *Argonaute* proteins. Plant Cell Physiol..

[32] Guo H.S., Xie Q., Fei J.F., Chua N.H. (2005). MicroRNA directs mRNA cleavage of the transcription factor NAC1 to downregulate auxin signals for *Arabidopsis* lateral root development. Plant Cell.

[33] Llave C., Xie Z., Kasschau K.D., Carrington J.C. (2002). Cleavage of *scarecrow-like* mRNA targets directed by a class of *Arabidopsis* miRNA. Science.

[34] Jagadeeswaran G., Saini A., Sunkar R. (2009). Biotic and abiotic stress down-regulate miR398 expression in *Arabidopsis*. Planta.

[35] Sunkar R., Kapoor A., Zhu J.K. (2006). Posttranscriptional induction of two Cu/Zn superoxide dismutase genes in *Arabidopsis* is mediated by downregulation of miR398 and important for oxidative stress tolerance. Plant Cell.

[36] Bouché N. (2010). New insights into miR398 functions in *Arabidopsis*. Plant Signal. Behav..

[37] Peragine A., Yoshikawa M., Wu G., Albrecht H.L., Poethig R.S. (2004). *SGS3* and *SGS2/SDE1/RDR6* are required for juvenile development and the production of *trans*-acting sirnas in *Arabidopsis*. Genes Dev..

[38] Vazquez F., Vaucheret H., Rajagopalan R., Lepers C., Gasciolli V., Mallory A.C., Hilbert J.L., Bartel D.P., Crete P. (2004). Endogenous *trans*-acting siRNAs regulate the accumulation of *Arabidopsis* mRNAs. Mol. Cell.

[39] Borsani O., Zhu J., Verslues P.E., Sunkar R., Zhu J.K. (2005). Endogenous siRNAs derived from a pair of natural *cis*-antisense transcripts regulate salt tolerance in *Arabidopsis*. Cell.

[40] Wang X.J., Gaasterland T., Chua N.H. (2005). Genome-wide prediction and identification of *cis*-natural antisense transcripts in *Arabidopsis thaliana*. Genome Biol..

[41] Zhang B., Pan X., Cobb G.P., Anderson T.A. (2006). Plant microRNA: A small regulatory molecule with big impact. Dev. Biol..

[42] Rajeswaran R., Aregger M., Zvereva A.S., Borah B.K., Gubaeva E.G., Pooggin M.M. (2012). Sequencing of RDR6-dependent double-stranded RNAs reveals novel features of plant siRNA biogenesis. Nucleic Acids Res..

[43] Zhai J., Jeong D.H., de Paoli E., Park S., Rosen B.D., Li Y., Gonzalez A.J., Yan Z., Kitto S.L., Grusak M.A. (2011). MicroRNAs as master regulators of the plant *NB-LRR* defense gene family via the production of phased, *trans*-acting siRNAs. Genes Dev..

[44] Fei Q., Xia R., Meyers B.C. (2013). Phased, secondary, small interfering RNAs in posttranscriptional regulatory networks. Plant Cell.

[45] Allen E., Xie Z., Gustafson A.M., Carrington J.C. (2005). MicroRNA-directed phasing during *trans*-acting siRNA biogenesis in plants. Cell.

[46] Axtell M.J., Jan C., Rajagopalan R., Bartel D.P. (2006). A two-hit trigger for siRNA biogenesis in plants. Cell.

[47] Rajagopalan R., Vaucheret H., Trejo J., Bartel D.P. (2006). A diverse and evolutionarily fluid set of microRNAs in *Arabidopsis thaliana*. Genes Dev..

[48] Fahlgren N., Montgomery T.A., Howell M.D., Allen E., Dvorak S.K., Alexander A.L., Carrington J.C. (2006). Regulation of *AUXIN RESPONSE FACTOR3* by *TAS3* ta-siRNA affects developmental timing and patterning in *Arabidopsis*. Curr. Biol..

[49] Moldovan D., Spriggs A., Yang J., Pogson B.J., Dennis E.S., Wilson I.W. (2010). Hypoxia-responsive microRNAs and *trans*-acting small interfering RNAs in *Arabidopsis*. J. Exp. Bot..

[50] Jen C.H., Michalopoulos I., Westhead D.R., Meyer P. (2005). Natural antisense transcripts with coding capacity in *Arabidopsis* may have a regulatory role that is not linked to double-stranded RNA degradation. Genome Biol..

[51] Zhang W., Zhou X., Xia J., Zhou X. (2012). Identification of microRNAs and natural antisense transcript-originated endogenous siRNAs from small-RNA deep sequencing data. Methods Mol. Biol..

[52] Jin H., Vacic V., Girke T., Lonardi S., Zhu J.K. (2008). Small RNAs and the regulation of *cis*-natural antisense transcripts in *Arabidopsis*. BMC Mol. Biol..

[53] Zhou X., Sunkar R., Jin H., Zhu J.K., Zhang W. (2009). Genome-wide identification and analysis of small RNAs originated from natural antisense transcripts in *Oryza sativa*. Genome Res..

[54] Zubko E., Meyer P. (2007). A natural antisense transcript of the *Petunia hybrida Sho* gene suggests a role for an antisense mechanism in cytokinin regulation. Plant J..

[55] Zhang X., Xia J., Lii Y.E., Barrera-Figueroa B.E., Zhou X., Gao S., Lu L., Niu D., Chen Z., Leung C. (2012). Genome-wide analysis of plant NAT-siRNAs reveals insights into their distribution, biogenesis and function. Genome Biol..

[56] Nozawa M., Miura S., Nei M. (2012). Origins and evolution of microRNA genes in plant species. Genome Biol. Evol..

[57] Merchan F., Boualem A., Crespi M., Frugier F. (2009). Plant polycistronic precursors containing non-homologous microRNAs target transcripts encoding functionally related proteins. Genome Biol..

[58] Bonnet E., Wuyts J., Rouze P., van de Peer Y. (2004). Detection of 91 potential conserved plant microRNAs in *Arabidopsis thaliana* and *Oryza sativa* identifies important target genes. Proc. Natl. Acad. Sci. USA.

[59] Lai E.C., Tomancak P., Williams R.W., Rubin G.M. (2003). Computational identification of *Drosophila* microRNA genes. Genome Biol..

[60] Sheng Y., Engstrom P.G., Lenhard B. (2007). Mammalian microRNA prediction through a support vector machine model of sequence and structure. PLoS ONE.

[61] Terai G., Komori T., Asai K., Kin T. (2007). miRRim: A novel system to find conserved miRNAs with high sensitivity and specificity. RNA.

[62] Wang X., Zhang J., Li F., Gu J., He T., Zhang X., Li Y. (2005). MicroRNA identification based on sequence and structure alignment. Bioinformatics.

[63] Dezulian T., Remmert M., Palatnik J.F., Weigel D., Huson D.H. (2006). Identification of plant microRNA homologs. Bioinformatics.

[64] Lim L.P., Glasner M.E., Yekta S., Burge C.B., Bartel D.P. (2003). Vertebrate microRNA genes. Science.

[65] Sewer A., Paul N., Landgraf P., Aravin A., Pfeffer S., Brownstein M.J., Tuschl T., van Nimwegen E., Zavolan M. (2005). Identification of clustered microRNAs using an *ab initio* prediction method. BMC Bioinform..

[66] Rice P., Longden I., Bleasby A. (2000). Emboss: The European molecular biology open software suite. Trends Genet..

[67] Jones-Rhoades M.W., Bartel D.P. (2004). Computational identification of plant microRNAs and their targets, including a stress-induced miRNA. Mol. Cell.

[68] Axtell M.J., Westholm J.O., Lai E.C. (2011). Vive la difference—Biogenesis ans evolution of microRNAs in plants and animals. Genome Biol..

[69] Thakur V., Wanchana S., Xu M., Bruskiewich R., Quick W.P., Mosig A., Zhu X.G. (2011). Characterization of statistical features for plant microRNA prediction. BMC Genom..

[70] Yang X., Li L. (2011). miRDeep-P: A computational tool for analyzing the microRNA transcriptome in plants. Bioinformatics.

[71] An J., Lai J., Sajjanhar A., Lehman M.L., Nelson C.C. (2014). miRPlant: An integrated tool for identification of plant miRNA from RNA sequencing data. BMC Bioinform..

[72] Mathelier A., Carbone A. (2010). MIReNA: Finding microRNAs with high accuracy and no learning at genome scale and from deep sequencing data. Bioinformatics.

[73] Friedlander M.R., Chen W., Adamidi C., Maaskola J., Einspanier R., Knespel S., Rajewsky N. (2008). Discovering microRNAs from deep sequencing data using miRDeep. Nat. Biotechnol..

[74] An J., Lai J., Lehman M.L., Nelson C.C. (2013). miRDeep*: An integrated application tool for miRNA identification from RNA sequencing data. Nucleic Acids Res..

[75] Meyers B.C., Axtell M.J., Bartel B., Bartel D.P., Baulcombe D., Bowman J.L., Cao X., Carrington J.C., Chen X., Green P.J. (2008). Criteria for annotation of plant microRNAs. Plant Cell.

[76] Lei J., Sun Y. (2014). miR-PREFeR: An accurate, fast and easy-to-use plant miRNA prediction tool using small RNA-seq data. Bioinformatics.

[77] Axtell M.J. (2013). ShortStack: Comprehensive annotation and quantification of small RNA genes. RNA.

[78] Chen H.-M., Li Y.-H., Wu S.-H. (2007). Bioinformatic prediction and experimental validation of a microRNA-directed tandem trans-acting siRNA cascade in *Arabidopsis*. Proc. Natl. Acad. Sci. USA.

[79] Dai X., Zhao P.X. (2008). pssRNAMiner: A plant short small RNA regulatory cascade analysis server. Nucleic Acids Res..

[80] Howell M.D., Fahlgren N., Chapman E.J., Cumbie J.S., Sullivan C.M., Givan S.A., Kasschau K.D., Carrington J.C. (2007). Genome-wide analysis of the RNA-dependent RNA polymerase6/dicer-like4 pathway in *Arabidopsis* reveals dependency on miRNA-and tasiRNA-directed targeting. Plant Cell.

[81] De Paoli E., Dorantes-Acosta A., Zhai J., Accerbi M., Jeong D.-H., Park S., Meyers B.C., Jorgensen R.A., Green P.J. (2009). Distinct extremely abundant siRNAs associated with cosuppression in petunia. RNA.

[82] McGinnis S., Madden T.L. (2004). BLAST: At the core of a powerful and diverse set of sequence analysis tools. Nucleic Acids Res..

[83] Smith T.F., Waterman M.S. (1981). Identification of common molecular subsequences. J. Mol. Biol..

[84] Fahlgren N., Howell M.D., Kasschau K.D., Chapman E.J., Sullivan C.M., Cumbie J.S., Givan S.A., Law T.F., Grant S.R., Dangl J.L. (2007). High-throughput sequencing of *Arabidopsis* microRNAs: Evidence for frequent birth and death of miRNA genes. PLoS ONE.

[85] Zhang Y. (2005). miRU: An automated plant miRNA target prediction server. Nucleic Acids Res..

[86] Li F., Orban R., Baker B. (2012). SoMART: A web server for plant miRNA, tasiRNA and target gene analysis. Plant J..

[87] Numnark S., Mhuantong W., Ingsriswang S., Wichadakul D. (2012). C-mii: A tool for plant miRNA and target identification. BMC Genom..

[88] Katara P., Gautam B., Kuntal H., Sharma V. (2010). Prediction of miRNA targets, affected proteins and their homologs in *Glycine max*. Bioinformation.

[89] Zhang C., Li G., Wang J., Fang J. (2012). Identification of *trans*-acting siRNAs and their regulatory cascades in grapevine. Bioinformatics.

[90] Liang G., Ai Q., Yu D. (2015). Uncovering miRNAs involved in crosstalk between nutrient deficienfies in *Arabidopsis*. Sci. Rep..

[91] Bonnet E., He Y., Billiau K., van de Peer Y. (2010). TAPIR, a web server for the prediction of plant microRNA targets, including target mimics. Bioinformatics.

[92] Dai X., Zhao P.X. (2011). psRNATarget: A plant small RNA target analysis server. Nucleic Acids Res..

[93] Milev I., Yahubyan G., Minkov I., Baev V. (2011). miRTour: Plant miRNA and target prediction tool. Bioinformation.

[94] Enright A.J., John B., Gaul U., Tuschl T., Sander C., Marks D.S. (2004). MicroRNA targets in *Drosophila*. Genome Biol..

[95] Krüger J., Rehmsmeier M. (2006). RNAhybrid: MicroRNA target prediction easy, fast and flexible. Nucleic Acids Res..

[96] Dai X., Zhuang Z., Zhao P.X. (2011). Computational analysis of miRNA targets in plants: Current status and challenges. Brief. Bioinform..

[97] Srivastava P.K., Moturu T.R., Pandey P., Baldwin I.T., Pandey S.P. (2014). A comparison of performance of plant miRNA target prediction tools and the characterization of features for genome-wide target prediction. BMC Genom..

[98] Addo-Quaye C., Eshoo T.W., Bartel D.P., Axtell M.J. (2008). Endogenous siRNA and miRNA targets identified by sequencing of the *Arabidopsis* degradome. Curr. Biol..

[99] German M.A., Luo S., Schroth G., Meyers B.C., Green P.J. (2009). Construction of parallel analysis of RNA ends (PARE) libraries for the study of cleaved miRNA targets and the RNA degradome. Nat. Protoc..

[100] Zhai J., Arikit S., Simon S.A., Kingham B.F., Meyers B.C. (2014). Rapid construction of parallel analysis of RNA end (PARE) libraries for Illumina sequencing. Methods.

[101] Song Q.-X., Liu Y.-F., Hu X.-Y., Zhang W.-K., Ma B., Chen S.-Y., Zhang J.-S. (2011). Identification of miRNAs and their target genes in developing soybean seeds by deep sequencing. BMC Plant Biol..

[102] Addo-Quaye C., Miller W., Axtell M.J. (2009). Cleaveland: A pipeline for using degradome data to find cleaved small RNA targets. Bioinformatics.

[103] Abdel-Ghany S.E., Pilon M. (2008). MicroRNA-mediated systemic down-regulation of copper protein expression in response to low copper availability in *Arabidopsis*. J. Biol. Chem..

[104] Fujii H., Chiou T.-J., Lin S.-I., Aung K., Zhu J.-K. (2005). A miRNA involved in phosphate-starvation response in *Arabidopsis*. Curr. Biol..

[105] Jia X., Wang W.-X., Ren L., Chen Q.-J., Mendu V., Willcut B., Dinkins R., Tang X., Tang G. (2009). Differential and dynamic regulation of miR398 in response to ABA and salt stress in *Populus tremula* and *Arabidopsis thaliana*. Plant Mol. Biol..

[106] Jagadeeswaran G., Li Y.F., Sunkar R. (2014). Redox signaling mediates the expression of a sulfate-deprivation-inducible microRNA395 in *Arabidopsis*. Plant J..

[107] Zhao M., Tai H., Sun S., Zhang F., Xu Y., Li W.-X. (2012). Cloning and characterization of maize miRNAs involved in responses to nitrogen deficiency. PLoS ONE.

[108] Hackenberg M., Gustafson P., Langridge P., Shi B.J. (2015). Differential expression of microRNAs and other small RNAs in barley between water and drought conditions. Plant Biotechnol. J..

[109] Kawashima C.G., Yoshimoto N., Maruyama-Nakashita A., Tsuchiya Y.N., Saito K., Takahashi H., Dalmay T. (2009). Sulphur starvation induces the expression of microRNA-395 and one of its target genes but in different cell types. Plant J..

[110] Yamasaki H., Abdel-Ghany S.E., Cohu C.M., Kobayashi Y., Shikanai T., Pilon M. (2007). Regulation of copper homeostasis by micro-RNA in *Arabidopsis*. J. Biol. Chem..

[111] Li W.X., Oono Y., Zhu J., He X.J., Wu J.M., Iida K., Lu X.Y., Cui X., Jin H., Zhu J.K. (2008). The *Arabidopsis* NYFA5 transcription factor is regulated transcriptionally and posttranscriptionally to promote drought resistance. Plant Cell.

[112] Zhou M., Li D., Li Z., Hu Q., Yang C., Zhu L., Luo H. (2013). Constitutive expression of a *miR319* gene alters plant development and enhances salt and drought tolerance in transgenic creeping bentgrass. Plant Physiol..

[113] Pall G.S., Hamilton A.J. (2008). Improved northern blot method for enhanced detection of small RNA. Nat. Protoc..

[114] Várallyay É., Burgyán J., Havelda Z. (2008). MicroRNA detection by northern blotting using locked nucleic acid probes. Nat. Protoc..

[115] Kim S.W., Li Z., Moore P.S., Monaghan A.P., Chang Y., Nichols M., John B. (2010). A sensitive non-radioactive northern blot method to detect small RNAs. Nucleic Acids Res..

[116] Fu H.J., Zhu J., Yang M., Zhang Z.Y., Tie Y., Jiang H., Sun Z.X., Zheng X.F. (2006). A novel method to monitor the expression of microRNAs. Mol. Biotechnol..

[117] Roberts T.C., Coenen-Stass A.M., Wood M.J. (2014). Assessment of RT-qPCR normalization strategies for accurate quantification of extracellular microRNAs in Murine Serum. PLoS ONE.

[118] Chen C., Ridzon D.A., Broomer A.J., Zhou Z., Lee D.H., Nguyen J.T., Barbisin M., Xu N.L., Mahuvakar V.R., Andersen M.R. (2005). Real-time quantification of microRNAs by stem-loop RT-PCR. Nucleic Acids Res..

[119] Moreau V., Voirin E., Paris C., Kotera M., Nothisen M., Rémy J.-S., Behr J.-P., Erbacher P., Lenne-Samuel N. (2009). Zip Nucleic Acids: New high affinity oligonucleotides as potent primers for PCR and reverse transcription. Nucleic Acids Res..

[120] Paris C., Moreau V., Deglane G., Voirin E., Erbacher P., Lenne-Samuel N. (2010). Zip nucleic acids are potent hydrolysis probes for quantitative PCR. Nucleic Acids Res..

[121] Javelle M., Timmermans M.C. (2012). *In situ* localization of small RNAs in plants by using LNA probes. Nat. Protoc..

[122] Begheldo M., Ditengou F., Cimoli G., Trevisan S., Quaggiotti S., Nonis A., Palme K., Ruperti B. (2013). Whole-mount *in situ* detection of microRNAs on *Arabidopsis* tissues using Zip Nucleic Acid probes. Anal. Biochem..

[123] Hsu R.J., Yang H.J., Tsai H.J. (2009). Labeled microRNA pull-down assay system: An experimental approach for high-throughput identification of microRNA-target mRNAs. Nucleic Acids Res.

[124] Jin H., Tuo W., Lian H., Liu Q., Zhu X.Q., Gao H. (2010). Strategies to identify microRNA targets: New advances. New Biotechnol..

[125] Kuhn D.E., Martin M.M., Feldman D.S., Terry A.V., Nuovo G.J., Elton T.S. (2008). Experimental validation of miRNA targets. Methods.

[126] Ghildiyal M., Zamore P.D. (2009). Small silencing RNAs: An expanding universe. Nat. Rev. Genet..

[127] Alonso J.M., Stepanova A.N., Leisse T.J., Kim C.J., Chen H., Shinn P., Stevenson D.K., Zimmerman J., Barajas P., Cheuk R. (2003). Genome-wide insertional mutagenesis of *Arabidopsis thaliana*. Science.

[128] Eamens A.L., Agius C., Smith N.A., Waterhouse P.M., Wang M.B. (2010). Efficient silencing of endogenous microRNAs using artificial microRNAs in *Arabidopsis thaliana*. Mol. Plant.

[129] Franco-Zorrilla J.M., Valli A., Todesco M., Mateos I., Puga M.I., Rubio-Somoza I., Leyva A., Weigel D., García J.A., Paz-Ares J. (2007). Target mimicry provides a new mechanism for regulation of microRNA activity. Nat. Genet..

